# Video mediastinoscopy-assisted superior mediastinal dissection in the treatment of thyroid carcinoma with mediastinal lymphadenopathy: preliminary results

**DOI:** 10.1186/s12893-021-01326-9

**Published:** 2021-08-18

**Authors:** Yuntao Song, Liang Dai, Guohui Xu, Tianxiao Wang, Wenbin Yu, Keneng Chen, Bin Zhang

**Affiliations:** 1 Key Laboratory of Carcinogenesis and Translational Research (Ministry of Education/Beijing), Department of Head and Neck Surgery, Peking University Cancer Hospital and Institute, 52 Fucheng Road, Haidian District, Beijing, China; 2 Key Laboratory of Carcinogenesis and Translational Research (Ministry of Education/Beijing), First Department of Thoracic Surgery, Peking University Cancer Hospital and Institute, Beijing, China

**Keywords:** Thyroid carcinoma, Video mediastinoscopy, Mediastinal lymph node metastases, Mediastinal dissection, Mediastinal lymphadenopathy

## Abstract

**Background:**

Mediastinal lymph node metastases (MLNM) are not rare in thyroid cancer, but their treatment has not been extensively studied. This study aimed to explore the preliminary application of video mediastinoscopy-assisted superior mediastinal dissection in the diagnosis and treatment of thyroid carcinoma with mediastinal lymphadenopathy.

**Materials and methods:**

We retrospectively reviewed the clinical pathologic data and short-term outcomes of thyroid cancer patients with suspicious MLNM treated with video mediastinoscopy-assisted mediastinal dissection at our institution from 2017 to 2020.

**Results:**

Nineteen patients were included: 14 with medullary thyroid carcinoma and five with papillary thyroid carcinoma. Superior mediastinal nodes were positive in nine (64.3%) patients with medullary thyroid carcinoma and in four (80.0%) patients with papillary carcinoma. No fatal bleeding occurred. There were three cases of temporary recurrent laryngeal nerve (RLN) palsy postoperatively, one of which was bilateral. Four patients had temporary hypocalcemia requiring supplementation, one had a chyle fistula, and one developed wound infection after the procedure. Postoperative serum molecular markers decreased in all patients. One patient died of cancer while the other 18 patients remained disease-free, with a median follow-up of 33 months.

**Conclusion:**

Video mediastinoscopy-assisted superior mediastinal dissection can be performed relatively safely in patients with suspicious MLNM. This diagnostic and therapeutic approach may help control locoregional recurrences.

## Introduction

The incidence of thyroid cancer (TC) has been continuously increasing worldwide during the past decades [1, 2]. Common TC categories, such as papillary thyroid carcinoma (PTC) and medullary thyroid carcinoma (MTC), tend to develop regional lymphatic metastasis [3], which is an important factor in predicting the structural recurrence of PTC [4] and is associated with decreased prognosis in MTC [5]. Surgical dissection is the first choice of treatment [6].

Lymph nodes involved in thyroid carcinoma could be classified into three regions: the central, lateral, and mediastinal compartments [7]. The central neck is the most commonly involved region, which is defined inferiorly by the superior sternal border [8]. Lymphatic tissue in this portion is in continuity with the superior mediastinum. Therefore, mediastinal lymph node metastases (MLNM) from TC are not uncommon. According to the literature, the incidence of MLNM was reported to range from 0.7 to 48.1% [9, 10]. Patients with mediastinal metastases have a poorer prognosis [11], and surgical extirpation is the preferred treatment for MLNM of thyroid cancer whenever possible.

Currently, there are two surgical approaches to treat MLNM. Transcervical approach is an extension of central compartment dissection, which is indicated for LNs located superior to innominate artery. While lower MLNM requires a more extensive operation. Sometimes, a partial-complete median sternotomy or thoracotomy is mandatory, which could potentially increase the risk of complications [12]. Ultrasound-guided fine needle aspiration cannot be easily done on mediastinal lymph nodes due to interference of bony structures of the chest wall. Consequently, it is difficult to confirm the enlarged mediastinal lymph node by pathology preoperatively, which thus leads to a diagnostic dilemma for physicians.

To minimize operative trauma in TC patients with suspected mediastinal metastasis, we explored a transcervical approach of video mediastinoscopy-assisted superior mediastinal dissection (VMSASMD), which has not been investigated in previous literature to the best of our knowledge. The goal of this study was to review a series of cases as preliminary communication demonstrating this technique.

## Materials and methods

A retrospective review was performed involving patients with thyroid carcinomas who underwent transcervical VMSASMD in the setting of suspicious mediastinal lymph nodes. Patients were treated between March 2017 and October 2020 at the Peking University Cancer Hospital. Demographic data, histology, incidence of mediastinal nodal metastasis, postoperative complications, and follow-up were reviewed. A Wilcoxon signed rank test was used to compare pre- and post-operative biomarkers. Data were analyzed using SPSS 22.0.

All patients required demonstration of suspicious MLNM on preoperative contrast-enhanced CT. CT features suggestive of metastasis included the presence of calcifications, central necrosis or cystic changes, and lymph nodes showing heterogeneous cortical enhancement or greater enhancement than the adjacent muscle [13]. Six patients underwent preoperative functional imaging examination, including ^99m^Tc-methoxyisobutylisonitrile (^99m^Tc-MIBI) single-photon emission computed tomography/computed tomography (SPECT/CT) or fluorine-18-deoxyglucose (FDG) positron emission tomography (PET), all with positive results. All patients underwent preoperative laryngoscopy and signed informed consent forms.

A multidisciplinary discussion was conducted by the head and neck surgeon, thoracic surgeon and radiologist. Indications for VMSASMD included: (1) PTC or MTC with suspected upper MLNM that are not amenable to remove through transcervical approach. (2) No major vascular involvement was found by imaging investigations. We routinely informed the patient of the possible complications of surgery and the possible instance that postoperative pathology may be negative. Other options, such as sternotomy, were also provided to the patient.

Close cooperation between head and neck surgeon and thoracic surgeon during surgery was important. All patients underwent dissection of pretracheal and paratracheal (level VI) lymphatic tissue through a transcervical incision above the sternal notch. If an intact or residual thyroid gland exists, a total or complemental thyroidectomy is performed, and if the lateral neck is clinically involved, it is dissected concurrently. Bilateral recurrent laryngeal nerves (RLNs) were routinely exposed with the assistance of intraoperative neuromonitoring (IONM, NIM-Response 3.0, Medtronic, Jacksonville, Florida, USA).

Standard open surgical instrumentation was used for cervical surgery. Part of the superior mediastinal LNs were taken together with the central neck specimen especially on the left side, which was superior to the innominate vein. The suprainnominate artery lymph nodes, also known as the level VII LNs, were resected through open approach as well.

**Fig. 1:**
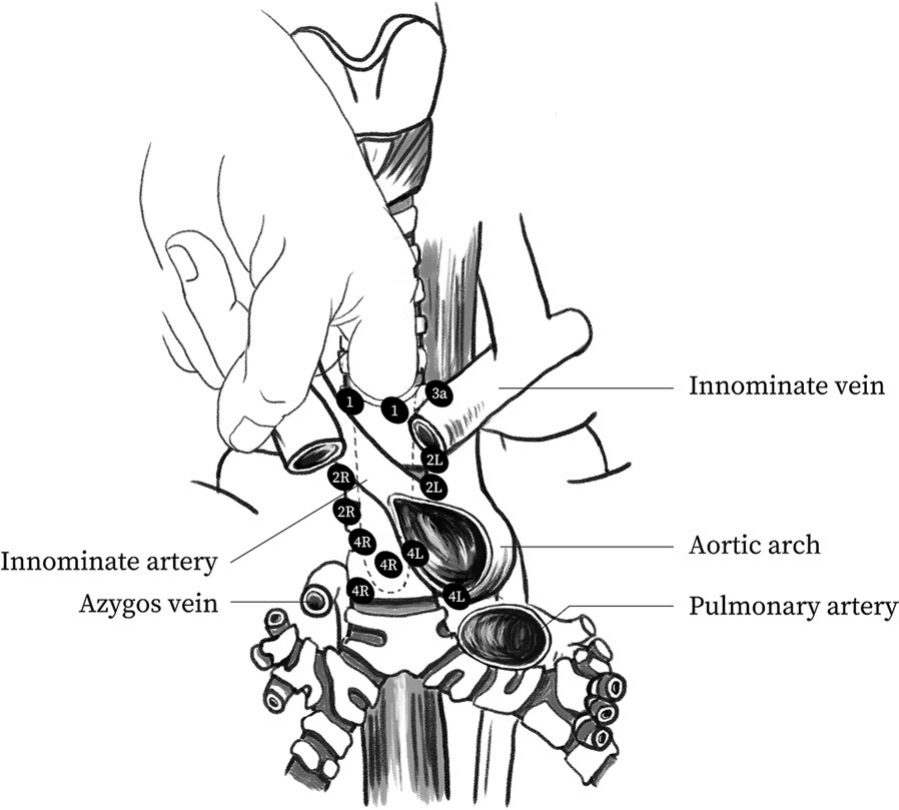
By separating the pretracheal fascia using the index finger, each compartment of the superior mediastinum was then exposed

Mediastinal dissection was performed by senior thoracic surgeons who were familiar with mediastinoscopic biopsy and sternotomy using video mediastinoscopy (Karl Storz, Tuttlingen, Germany). The equipment for immediate sternotomy or thoracotomy was also prepared in case of intraoperative conversion. The surgeon stood on the cranial side of the patient and the video monitor was placed on the caudal side. Through routine cervical thyroid incision, the thymus is separated from the trachea. The index finger followed the trachea and broke the pretracheal fascia (Fig. 1). Then, the scope is introduced with the blades closed.

**Fig. 2:**
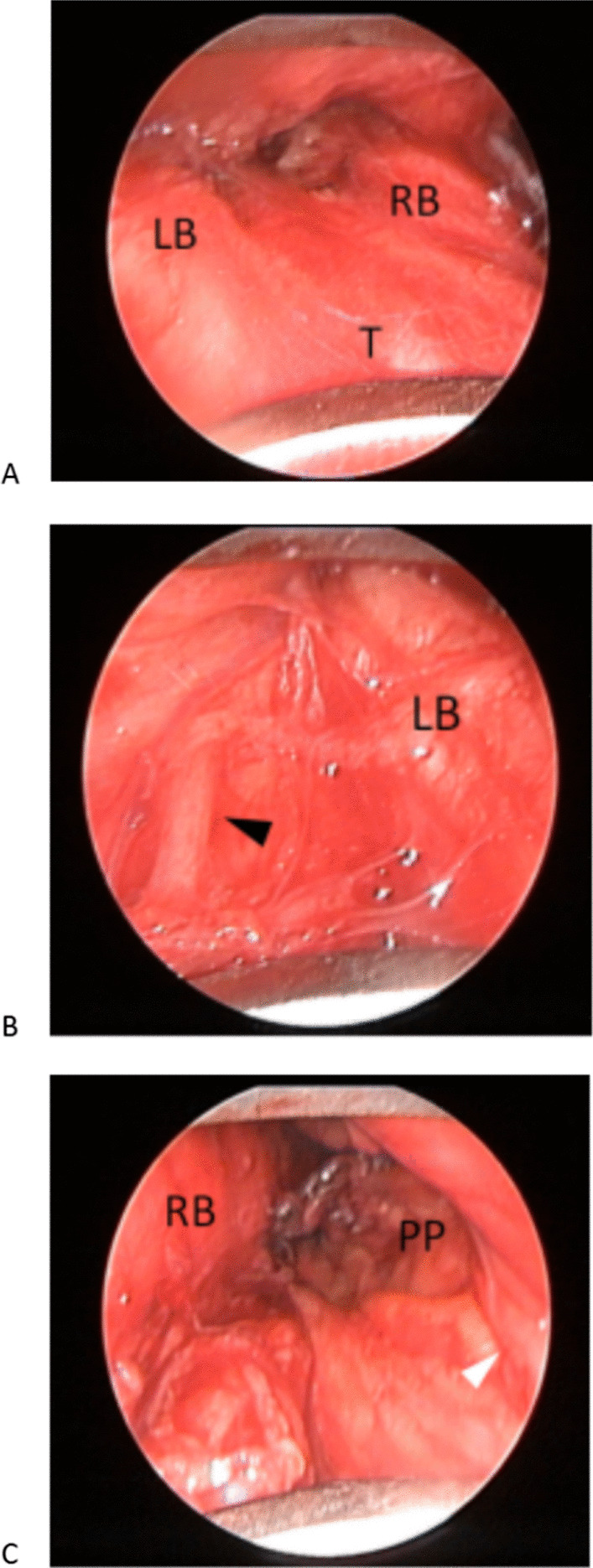
**A** Central compartment, **B** Left compartment; **C** Right compartment. *T* trachea, *LB* left main bronchus, *RB* right main bronchus, *PP* parietal pleura; black arrow, left vagus nerve; white arrow, right vagus nerve

The right pulmonary artery was separated, the scope blades were spread, the right and left tracheobronchial angles were identified (Fig. 2A), and the axis of the scope was twisted to the left. The left recurrent laryngeal nerve was identified and exposed (Fig. 2B). A thorough dissection of the adipose tissue of lymph nodes in station 4L was performed while preserving the function of the left recurrent nerve. Routine dissection of the subcarinal space is not necessary unless lymph nodes in that area are suspected. The right compartment is the largest compartment. The scope was fixed from the tracheal axis to the right, and the lymph nodes were dissected away from the innominate artery and vein, superior vena cava, and right parietal pleura (Fig. 2C). The mediastinal lymph node specimens were divided and labeled according to the criteria for lung cancer [14].

## Results

Nineteen patients with thyroid cancer were included, wherein 14 had MTC and 5 had PTC, all of the MTC patients were sporadic. Four (21.1%) patients were initially treated, including three with MTC and one with PTC, while others underwent re-operation. The median age of the patients was 39 years (range 15–65 years). Eight patients were women (42.1%) and 11 were men (57.9%). Patient demographics, tumor stages, and treatments are listed in Table 1.

**Table 1:** Characteristics of the patients

	MTC (n = 14)	PTC (n = 5)
Gender		
Male	8	3
Female	6	2
Age (y) median (range)	38 (15–65)	54 (29–63)
AJCC stage		
I	1	2
II	0	3
IVA	13	0
Frequency of operation		
Initial surgery	3	1
Reoperation	11	4
Simultaneous operation		
TT + CND	1	0
TT + CND + LND	4	2
CND	2	1
CND + LND	7	2
MLNM		
Yes	9	4
No	5	1
Outcome		
Disease-free	14	4
Death	0	1

*MTC* medullary thyroid carcinoma, *PTC* papillary thyroid carcinoma, *TT* total thyroidectomy, *CND* central lymph node dissection, *LND* lateral lymph node dissection, *MLNM* mediastinal lymph node metastasis, *ETE* extrathyroidal extension

All patients underwent VMSASMD successfully without intraoperative conversion to sternotomy or thoracotomy. No major vessel injury occurred during superior mediastinal dissection. The mean operation time was 206 min (SD, 58 min; range 100–320 min). The mean blood loss was 65 ml (SD, 30 ml; range 20–100-ml) and no blood transfusion was required. Preoperative laryngoscopy revealed unilateral paralyzed vocal motility in two patients with MTC, whose recurrent laryngeal nerve (RLN) in the affected side was invaded by the tumor and was resected. For other patients with normal pre-resection intraoperative electromyographic (EMG) signals, two developed unilateral temporary RLN palsy and recovered within 1 to 4 months. One patient developed bilateral RLN palsy during surgery and required prophylactic tracheotomy. The tube was removed 1 week later and there was normalization of voice quality 3 months after surgery. The laryngoscope showed normal activity of the bilateral vocal cords (Table 2).

**Table 2:** Prevalence of complications and their relationship with VMSASMD

Complications	No. (%)	VMSASMD-related
RLN palsy		
Transient	3^a^ (15.8)	3
Permanent	2^b^ (10.5)	0
Hypocalcemia		
Transient	4 (21.1)	0
Permanent	1^c^ (5.3)	0
Chyle fistula	1 (5.3)	0
Infection	1 (5.3)	0

*VMSASMD* video mediastinoscopy assisted superior mediastinal dissection

^a^One patient had bilateral RLN palsy

^b^RLN resection due to tumor invasion

^c^One permanent hypoparathyroidism before operation

One patient with recurrent MTC had permanent hypoparathyroidism before surgery but had no significant change in parathyroid hormone (PTH) levels after surgery (5.2 pg/ml vs. 5.1 pg/ml). Among the patients, four experienced temporary hypoparathyroidism, with three of them asymptomatic. Their PTH levels were all restored 6 months after the operation. The median total drainage volume was 357 ml (quartile: 175, 740 ml; range 25–9160 ml). One patient developed a chyle fistula (CF) after the procedure and was referred for surgery (ligature of the thoracic duct under thoracoscopy) after conservative treatment failed. One patient had a postoperative wound infection, which healed after debridement and antibiotic use. The median hospitalization time was 10 days (interquartile range—9, 14 days; and range 7–38 day).

Overall, superior mediastinal disease was present in 13 (68.4%) out of the 19 patients. MLNM was found in 9 of 14 (64.3%) patients with medullary carcinoma and in 4 (80.0%) out of 5 patients with papillary carcinoma. Six patients with positive results on functional imaging (^99m^Tc-MIBI SPECT/CT in 1 patient with MTC, PET/CT in 3 patients with MTC, and 2 patients with PTC) all had MLNM upon pathologic examination. The number of lymph nodes harvested from the superior mediastinum was 229 in total and 11.8 on average, with 43 of 229 being malignant (18.8%). Lymph nodes were distributed at levels 2R, 4R, 2L, 4L, 3A, and at the subcarina (level 7) (Table 3). Extracapsular extension was present in 10 (76.9%) out of 13 MLNM patients, including 8 (88.9%) of 9 with MTC and 2 (50.0%) of 4 with PTC.

**Table 3:** Distribution of metastatic LNs in superior mediastinum

Level	Number of patients undergoing dissection	Number of patients metastasis
2R	19	11
2L	2	0
3A	5	3
4R	18	3
4L	4	0
7 (subcarina)	1	1

**Fig. 3:**
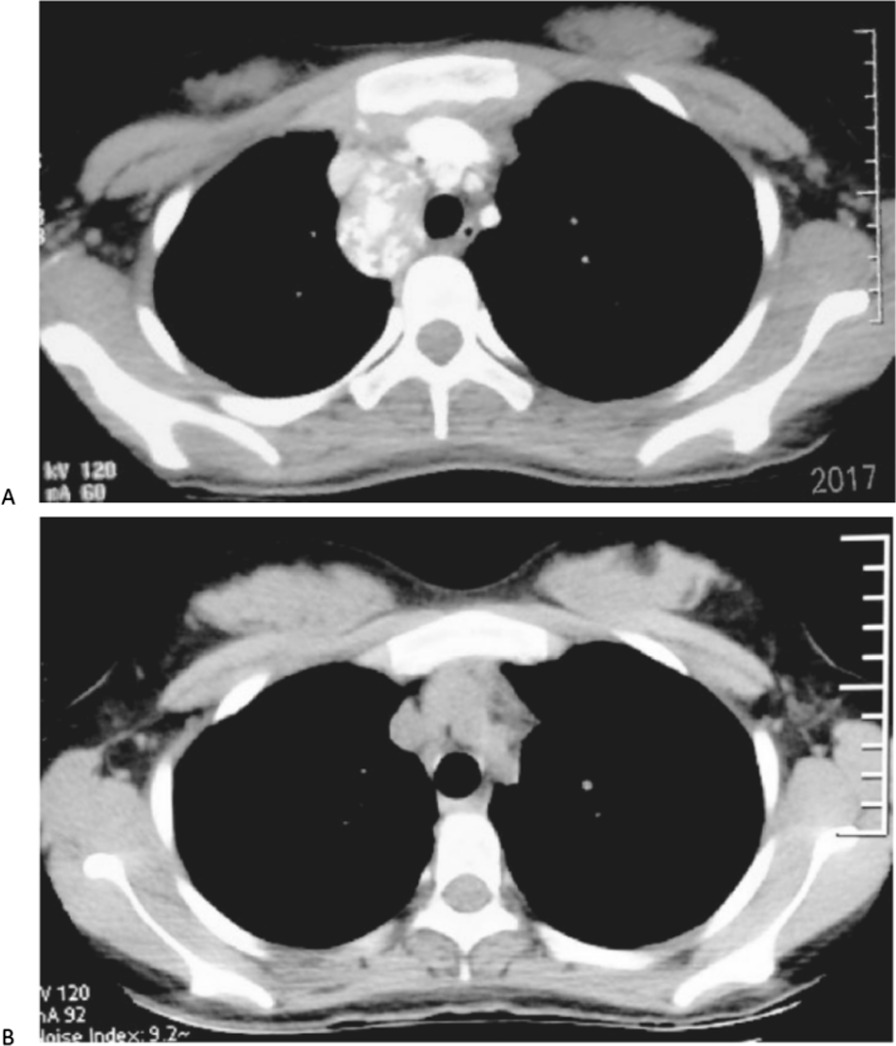
A 15-year-old patient with recurrent MTC. **A** Preoperative findings of suspicious lymph nodes in the right upper mediastinal compartments upon enhanced CT scan. **B** Postoperative image 3 years after video mediastinoscopy-assisted superior mediastinal dissection and central and lateral neck dissection was performed

The median follow-up time was 33 months (range 3–47 months). One patient with papillary thyroid carcinoma with lung metastasis died of cancer 16 months postoperatively while the others are currently alive. At the last follow-up, clinical examination and radiographic studies were negative for recurrent tumors in all patients (Fig. 3). In MTC patients, the median preoperative serum calcitonin (CT) level was 1444.0 pg/ml (quartile: 814.4, 2000 pg/ml; range 162.4 pg/ml to > 2000 pg/ml). Because the upper limit of calcitonin level was 2000 pg/ml in this institute’s laboratory, the serum calcitonin level was calculated to be 2000 pg/ml. The median post-operative serum calcitonin level was 168.3 pg/ml (quartile: 40.4, 661.0 pg/ml; range 0.5 pg/ml to > 2000 pg/ml). The value significantly decreased compared with the preoperative value (p = 0.001). There was only one patient whose serum calcitonin level was beyond the upper limit before and after surgery, when her blood test was performed in another hospital, the serum calcitonin level was > 20,000 pg/ml preoperatively and 4300 pg/ml postoperatively. For these patients, calcitonin levels remained stable postoperatively, with only one patient in the normal range. All five patients with PTC received radioiodine therapy before or after VMSASMD. Postoperative serum thyroglobulin (Tg) levels decreased in all living patients to < 2 ng/ml, while serum thyroglobulin antibody (Tg-Ab) levels were normal.

## Discussion

Lymph node metastases are common in thyroid cancer patients, with a high incidence of both occult and overt metastases [15, 16]. Most studies have focused on central and lateral lymph node metastases, but the incidence of MLNM and the extent of mediastinal lymph node dissection are not clearly defined.

Although MTC and PTC have different pathological origins and prognoses, surgical techniques for the management of primary tumors and regional metastases are the same, which is the primary focus of our research. The majority of our cohort had MTC because it has a worse prognosis and had no established adjuvant therapy, thus requiring more extensive surgery. Sometimes, elective dissection may be recommended.

Most experts agree that sternotomy with mediastinal dissection should be reserved for PTC and MTC patients with imaging evidence of mediastinal disease [4, 17]. However, radiological evaluation has a low accuracy for detecting mediastinal nodal disease. In a study of 94 patients with highly suspected MLNM who underwent mediastinal lymph node dissection, 13 (13.8%) patients were pathologically negative [10]. Ducic et al. [18] performed transcervical elective superior mediastinal dissection in certain patients with papillary, medullary, and anaplastic thyroid carcinomas and found that 19 of 31 (61.3%) were positive even without overt mediastinal adenopathy during preoperative evaluation. Sugenoya et al. [9] found mediastinal lymph node metastases in 10 of 21 patients (48%) with advanced differentiated thyroid carcinoma after mediastinal dissection through partial midline sternotomy. In particular, for previously untreated MTC, if pretherapeutic basal calcitonin levels were greater than 500 pg/ml, mediastinal dissection was strongly recommended [19]. Because of the difficulty in doing a biopsy of mediastinal lymph nodes, there will always be some patients with negative postoperative pathological results. This proportion was 31.6% in the present study. Obviously, it is not worthwhile for patients without metastasis to undergo such an operation, which can cause excessive trauma.

There are two main approaches to mediastinal lymph node dissection: the transcervical or the transsternal procedure. The transcervical approach is more convenient but has a limited surgical field. Sternotomy and partial sternotomy are most frequently used for mediastinal dissection since they can offer greater exposure, but they also have greater surgical invasiveness [12, 20, 21]. Video mediastinoscopy is a minimally invasive strategy which is seldom used in the treatment of MLNM from thyroid cancer. Mediastinoscopy was initially used in the mediastinal staging of lung cancer [22]. Video mediastinoscopy (VMS) enables the surgeon to operate bimanually as in open surgery. The superior mediastinum is entered through a transcervical incision and visualization of the area caudal to the subcarinal lymph nodes is facilitated and flashed on the video screen [23, 24].

There is a lack of studies describing the terminology and classification of mediastinal lymph node dissection; therefore, we adopted the mediastinal division compartments of thoracic surgery. We found that level 2R was the most commonly involved compartment, probably because the inferior border of the central compartment is defined as the innominate artery on the right and the corresponding axial plane on the left [25], while the boundary of levels 2R and 2L is the left margin of the trachea. As a result, the extent of level 2R is larger than 2L, and the latter tends to be contiguous with the left central neck compartment and removed together with the central neck dissection specimen.

In our experience, lymph nodes can be easily identified and resected without compromising the adjacent tissues under VMS. This is mainly because lymph nodes with thyroid cancer metastasis usually have a smooth capsule and are well demarcated from the surrounding tissues. However, extreme caution should be exercised when grasping the lymph node tissue. Gentle traction must also be used while dissecting the surrounding structures to avoid fatal bleeding [26].

The postoperative complication rate was relatively higher; some had a prolonged time of hospitalization, but there was no development of any avoidable permanent sequelae. Among the complications, one patient developed bilateral RLN palsy and required tracheostomy. Bilateral RLN injury is a rare complication. It is reported to occur in one out of 1000 cases following total thyroidectomy in a specialized thyroid unit [27]. The possible reasons in our cohort may be that the surgeons were inexperienced in the first few cases of bilateral mediastinal dissection. A muscle relaxant was used during the mediastinal procedure to avoid bucking or movement of the patients; hence, IONM was temporarily not applied. With technical improvements, the incidence of RLN injury has been reduced afterward. One patient developed a chyle leak, which is not a rare complication of neck dissection. In this case, it is not known whether thoracic duct injury occurred in the neck or chest. Therefore, we performed more active surgical management to prevent fatal mediastinal infection.

Considering the high proportion of revision surgeries (15/19, 78.9%) in our cohort, the safety of VMSASMD was acceptable. A study of mediastinal lymph node dissection for thyroid carcinoma through a sternotomy or partial sternotomy approach revealed a postoperative complication rate of 38.2% (13/34), which was significantly higher than that of the transcervical approach (28.4%, 25/88) [21]. Mediastinal operation-associated complications associated with the sternotomy approach, such as pleural effusion, mediastinal infection, and superior vena cava rupture, were not observed in our study.

The short follow-up period was a deficiency of this study, especially for indolent tumors like PTC and MTC. Calcitonin (CT) is a sensitive and specific marker for the persistence or recurrence of MTC and has been used to determine the success of operations for many years. Previous reports have shown that a decrease in CT was not ideal in patients undergoing reoperation. Moley et al. [28] performed 35 repeat neck explorations and microdissections in 32 patients and found that in 10 cases, the CT levels did not decrease. Even in experienced hands, reoperation on selected patients can only yield biochemical cure rates of 30–40% [29]. It may be lower in patients with suspected mediastinal metastasis. Nevertheless, many patients with persistently high levels of CT after surgery continue to live without evidence of disease for many years because of the indolent pattern of the tumor. The serum CT levels of MTC patients in our cohort all decreased after surgery, but only one of them who underwent initial surgery had normalization of CT levels. None of the MTC patients had radiologic recurrence, but a longer follow-up is needed for further research.

The prognosis of PTC patients with MLNM has rarely been studied. Moritani et al. [11] investigated the impact of mediastinal metastases on the prognosis of PTC based on a mean 10.5-year follow-up of 488 patients. They found significant differences in disease-free survival (DFS) between patients with and without mediastinal metastases. One of the four patients with MLNM of PTC who died of cancer in our study had pulmonary metastases before surgery. This may dispute the significance of palliative mediastinal lymph node dissection in patients with distant metastasis, but the number of cases are too small to provide meaningful statistical results.

## Conclusions

Video mediastinoscopy-assisted superior mediastinal dissection can be performed safely in patients with MLNM without sternotomy, especially when malignancy is uncertain. This diagnostic and therapeutic approach may help control locoregional recurrences, and further studies are necessary to determine the impact of MLNM on the long-term prognosis of thyroid cancer patients.

## Data Availability

The datasets used and analyzed during the current study are available from the corresponding author upon reasonable request.
